# StABI5 Involved in the Regulation of Chloroplast Development and Photosynthesis in Potato

**DOI:** 10.3390/ijms21031068

**Published:** 2020-02-06

**Authors:** Tingting Zhu, Linxuan Li, Li Feng, Maozhi Ren

**Affiliations:** 1 Institute of Urban Agriculture, Chinese Academy of Agricultural Sciences, Chengdu 610213, China; 20172602005t@cqu.edu.cn (T.Z.); lilinxuan@caas.cn (L.L.); 18202321576@163.com (L.F.); 2 School of Life Sciences, Chongqing University, Chongqing 400045, China; 3 Zhengzhou Research Base, State Key Laboratory of Cotton Biology, Zhengzhou University, Zhengzhou 450000, China

**Keywords:** chloroplast development, chlorophyll, photosynthesis, potato, StABI5

## Abstract

Abscisic acid (ABA) insensitive 5 (ABI5)—a core transcription factor of the ABA signaling pathway—is a basic leucine zipper transcription factor that plays a key role in the regulation of seed germination and early seedling growth. ABI5 interacts with other phytohormone signals to regulate plant growth and development, and stress responses in *Arabidopsis*, but little is known about the functions of ABI5 in potatoes. Here, we find that StABI5 is involved in the regulation of chloroplast development and photosynthesis. Genetic analysis indicates that *StABI5* overexpression transgenic potato lines accelerate dark-induced leaf yellowing and senescence. The chlorophyll contents of overexpressed *StABI5* transgenic potato lines were significantly decreased in comparison to those of wild-type Desiree potatoes under dark conditions. Additionally, the RNA-sequencing (RNA-seq) analysis shows that many metabolic processes are changed in overexpressed *StABI5* transgenic potatoes. Most of the genes involved in photosynthesis and carbon fixation are significantly down-regulated, especially the chlorophyll a-b binding protein, photosystem I, and photosystem II. These observations indicate that StABI5 negatively regulates chloroplast development and photosynthesis, and provides some insights into the functions of StABI5 in regard to potato growth.

## 1. Introduction

Abscisic acid (ABA) is a pivotal phytohormone that regulates plant growth and development as well as abiotic and biotic stress responses [1,2,3]. ABA insensitive 5 (ABI5), which is a basic leucine zipper (bZIP) transcription factor, plays important roles in core ABA signaling by controlling seed dormancy, germination, plant growth, and stress responses. The *abi5* mutant was originally obtained by screening ABA-insensitive phenotype from an *Arabidopsis* T-DNA mutant library [4]. The *abi5* mutant has pleiotropic defects in ABA response, including decreased sensitivity to ABA-induced inhibition of germination and the altered expression of ABA-regulated genes [5,6], which suggests the role of ABI5 in abiotic stress response. ABI5 directly binds to the ABA-responsive element (ABRE) within the promoter regions of target genes containing *early methionine-labeled 1* (*EM1*) and *EM6* to regulate their expression. In the *abi5* mutant, the expression levels of *EM1* and *EM6* have been shown to be significantly down-regulated, and displayed a phenotype that is insensitive to ABA and NaCl [6]. Furthermore, ABI5 has an important function in seed germination by regulating the expression of *polygalacturonase inhibiting protein 1* (*PGIP1*) and *PGIP2* genes. ABI5 inhibits polygalacturonase activity by inducing the expression of *PGIP1* and *PGIP2* genes, which blocks seed coat rupture and inhibits seed germination [7]. In addition, recent studies show that ABI5 interacts with other plant hormone signals to regulate seed germination. Jasmonate-ZIM domain (JAZs), which are negative regulatory proteins of jasmonic acid (JA) signaling, repress the transcriptional activity of ABI5 and modulate seed germination in bread wheat and *Arabidopsis* [8]. The brassinosteroid insensitive 2 (BIN2) kinase, a key repressor of brassinosteroid (BR) signaling, has been shown to phosphorylate and stabilize ABI5 during seed germination [9]. By contrast, the brassinazole resistant 1 (BZR1) and BR insensitive 1-EMS-suppressor 1 (BES1) transcription factors of the BR signaling pathway can suppress ABI5 transcriptional activity and promote seed germination in *Arabidopsis* [10,11]. Similarly, DELLA proteins of gibberellic acid (GA) signaling promote the transcriptional activity of ABI5 to inhibit seed germination and postgerminative growth, whereas the transcription factor inducer of CBF expression 1 (ICE1) interferes with the transcriptional function of ABI5 and promotes seed germination [12,13].

In addition to seed germination, ABI5 also regulates postgerminative growth and vegetative tissue. ABI5 is not only expressed in root tips, stems and leaf veins, but also in the edges of old leaves and flowers. Diacylglycerol acyltransferase 1 (DGAT1), a rate-limiting enzyme of triacylglycerol biosynthesis, has been shown to be regulated by ABI5 to accumulate triacylglycerol in plant seedlings under stress [14]. Furthermore, ABI5 is also involved in regulating plant photosynthesis in *Arabidopsis*. For example, the *staygreen 1* (*SGR1*) and *non-yellow coloring 1* (*NYC1*) genes that are responsible for chlorophyll catabolism—containing an ABRE element in their promoters—are positively regulated by ABI5, reflecting that ABI5 is a negative regulator of photosynthesis through the activation of chlorophyll degradation [15]. The late embryogenesis abundant (LEA) protein ABA-response protein (ABR) is regulated by ABI5 and is involved in dark-induced leaf senescence, implying that ABI5 plays a positive role in this process through the negative regulation of photosynthesis in *Arabidopsis* [16]. Besides, lateral root formation is also regulated by ABI5. ABA induces *ABI5* expression in the lateral root tips, and the effect of ABA- and nitrate-mediated lateral root growth inhibition is significantly reduced in *abi4* and *abi5* mutants [17].

The regulation of *ABI5* expression is complex and is mediated by multiple transcription factors and proteins. The ABI3 and ABI4 transcription factors of ABA signaling positively regulate the expression of *ABI5* during both seed germination and postgerminative growth [18,19]. Interestingly, ABI5 also directly binds to its own promoter through the G-box motif and activates the expression of itself [20]. The light-signaling transcription factor elongated hypocotyl 5 (HY5) binds to the promoter of *ABI5* gene and acts as a transcriptional activator of *ABI5* expression [21]. However, the B-box 21 (BBX21) is a negative regulator of *ABI5* expression—interfering with the binding of HY5 and ABI5 to the *ABI5* promoter [20]. Stress-activated transcription factor MYB96, a negative regulator of lateral root formation, activates the expression of *ABI5*, which supports the role of ABI5 in the inhibition of lateral root growth [17,22]. By contrast, the other transcription factor MYB7 negatively regulates *ABI5* expression during seed germination [23]. Moreover, WRKY-domain transcription factors, such as WRKY18, WRKY40 and WRKY60, bind to the W-box motif within the promoter of *ABI5* gene and thus repress *ABI5* expression during post-germinative growth [24]. The phosphorylation and stability of the ABI5 protein is regulated by its interaction with other proteins. In addition to SNF1-related protein kinases (SnRK2s), calcineurin B-like interacting protein kinase 26 (CIPK26), CIPK11 and BIN2 also phosphorylate ABI5 in vitro [9,25,26,27]. ABI5 dephosphorylation and destabilization depends on protein phosphate 2A (PP2A) and two catalytic subunits of PP6 phosphatase [28,29]. Additionally, ABI5 ubiquitination and 26S proteasomal degradation can be mediated by KEEP ON GOING (KEG) and CULLIN 4-based E3 ubiquitin ligases [30,31,32]. These studies show that ABI5 activity and stability are regulated by multiple posttranslational modifications.

However, despite the abundant evidence of the involvement of ABI5 in ABA and other signaling pathways during seed germination and post-germinative growth in *Arabidopsis*, details of the transcriptional regulation of StABI5 largely remain unknown in potato. In this study, the function of StABI5 in regulating chloroplast development and photosynthesis was identified. Genetic analysis indicated that *StABI5* overexpression potato lines accelerated dark-induced leaf yellowing and senescence. Additionally, RNA-seq experiments were performed to test the functions of StABI5 in potato. The RNA-seq analysis showed that ribosomes, photosynthesis, and many metabolic processes were changed in *StABI5* overexpression potatoes. Importantly, most chlorophyll a-b binding proteins and carbon fixation genes were down-regulated in *StABI5* overexpression potatoes, implying that StABI5 is involved in the negative regulation of photosynthesis. Taken together, our results showed that StABI5 played important roles in the regulation of chloroplast development and photosynthesis in potato.

## 2. Results

### 2.1. ABI5 Regulates Chlorophyll Catabolism Under Dark Conditions

**Figure 1:**
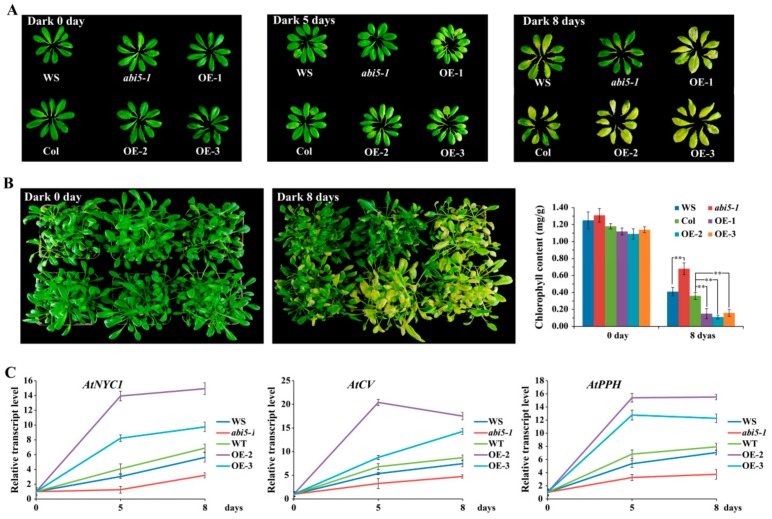
AtABI5 regulates chlorophyll catabolism in *Arabidopsis*. (**A**) Phenotype of the *abi5-1* mutant and overexpressed *AtABI5* (OE) transgenic *Arabidopsis* leaves after 0, 5, and 8 days of dark treatment, respectively. (**B**) Phenotype and chlorophyll content of the *abi5-1* mutant and overexpressed *AtABI5* (OE) transgenic lines after 0 and 8 days of dark treatment, respectively. The data represent the mean ± SD of 3 independent experiments. Asterisks denote Student’s t-test significant differences as compared with Col (** *P* < 0.01). (**C**) Transcription levels of chlorophyll catabolism-related genes in *Arabidopsis* after dark treatment. The data represents the mean ± SD of 3 independent experiments.

The transcription factor ABI5 not only plays an indispensable role in the ABA signaling pathway, but also plays a key role in seed maturation, germination, and early seedling growth regulation [8,33]. In addition, ABI5 also acts as an integrator for ABA and other plant hormone signals. Recent studies have shown that ABI5 is involved in photosynthesis by regulating chlorophyll metabolism [15,34,35]. To further analyze the function of ABI5 in chlorophyll metabolism, we treated the *abi5-1* mutant and overexpressed *AtABI5* transgenic *Arabidopsis* plants and leaves for 0, 5, and 8 days under dark conditions. The results showed that dark treatment can accelerate senescence and yellowing of leaves in *Arabidopsis* (Figure 1A,B). The *abi5-1* mutant reduced leaf senescence and yellowing as compared to the wild type (WS), but overexpressed *AtABI5* transgenic *Arabidopsis* accelerated this process. Furthermore, the chlorophyll content of the overexpressed *AtABI5* transgenic lines was significantly lower than the Columbia (Col), whereas the *abi5-1* mutant was higher than WS, which further verified the above results. To determine whether ABI5 promoted the expression of chlorophyll catabolism-related genes under dark conditions, we tested the transcription levels of chlorophyll catabolism-related genes *AtNYC1*, *AtCV,* and *AtPPH* after dark treatment in *Arabidopsis*. These results showed that overexpressed *AtABI5* transgenic *Arabidopsis* lines significantly upregulated the transcription levels of *AtNYC1*, *AtCV,* and *AtPPH* genes as compared to Col after dark treatment, while the *abi5-1* mutant attenuated the up-regulation of these genes, which in turn delayed the senescence and yellowing of leaves (Figure 1C).

### 2.2. Overexpressed StABI5 Potato Lines Accelerate Dark-Induced Leaf Yellowing and Senescence

Potato (*Solanum tuberosum*) belongs to the *Solanaceae* family, whose tubers are edible and is the fourth largest food crop in the world after wheat, rice, and corn. In addition, it is also a kind of cash crop with significant advantages. Therefore, analyzing the roles of StABI5 in potato has important practical significance and economic value. A BLASTp analysis of the potato genome database (http://plants.ensembl.org/Solanum_tuberosum/Info/Index) was performed using the AtABI5 protein as reference. The BLASTp analysis results showed that the protein sequence of the *PGSC0003DMG400002660* gene shared the highest similarity of 49.26% with AtABI5, so we named the gene as *StABI5*. The *StABI5* gene sequence contains 4 exons and 3 introns, which encodes 428 amino acid residues with molecular mass of 46 kDa (Figure S1A). Phylogenetic analysis of StABI5 and other species of ABI5 proteins showed that the evolutionary relationship between potato StABI5 and tomato SlABI5 is the closest, and it is the furthest from rice OsABI5 in regard to evolution (Figure S1B). Consistent with the *Arabidopsis* AtABI5 protein [33], StABI5 also contains conserved C1, C2, C3, bZIP, and C4 domains (Figure S1C).

**Figure 2:**
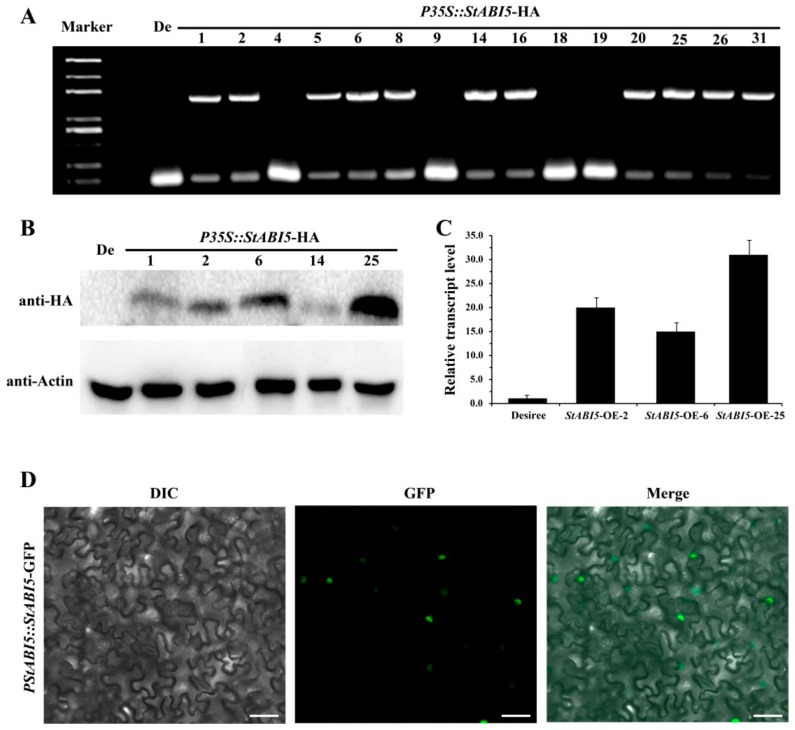
Identification and subcellular localization of *StABI5* transgenic potatoes. (**A**) Identification of *StABI5* transgenic potato lines by leaf PCR. The amplified PCR product was 1547 bp when using primers P35S F and *StABI5* R. De: Desiree. (**B**) Western blot analysis of StABI5 protein expression in *StABI5* transgenic potato lines. (**C**) The transcription level of *StABI5* in *StABI5* transgenic potato lines obtained by qRT-PCR. *StACTIN58* was used as an internal control. (**D**) Subcellular localization of StABI5 in tobacco (*Nicotiana tabacum*). Tobacco transiently expressed *Agrobacterium* strain containing the *PStABI5*::*StABI5*-*GFP* vector, and the localization of GFP was observed by laser confocal microscopy. Bars, 50 μm.

To further analyze the functions of StABI5 in potato, we constructed the *StABI5* overexpression vectors *P35S::StABI5-HA* and *PStABI5::StABI5-GFP*. Through agrobacterium-mediated genetic transformation technology, we obtained transgenic potato lines with the *P35S::StABI5-HA* overexpression vector. We preliminarily identified transgenic lines containing the target vector by leaf PCR using primers P35S F and *StABI5* R, and a total of 11 transgenic lines were identified (Figure 2A). Then, the protein expression of StABI5 in *P35S::StABI5-HA* transgenic potato lines was further detected by Western blot analysis. The results showed that the *P35S::StABI5-HA-25* transgenic potato line had the highest protein expression level of StABI5, followed by the *P35S::StABI5-HA-2* and *P35S::StABI5-HA-6* lines (Figure 2B). Correspondingly, the *StABI5* transcription level of the *P35S::StABI5-HA-25* (StABI5-OE-25) line was also the highest among *StABI5* transgenic potato lines (Figure 2C). To clarify the subcellular localization of StABI5 protein, we transiently expressed *Agrobacterium* strain containing the *PStABI5*::*StABI5*-*GFP* vector and observed the localization of GFP in tobacco. The results indicated that StABI5 was localized in the nucleus in tobacco (Figure 2D).

**Figure 3:**
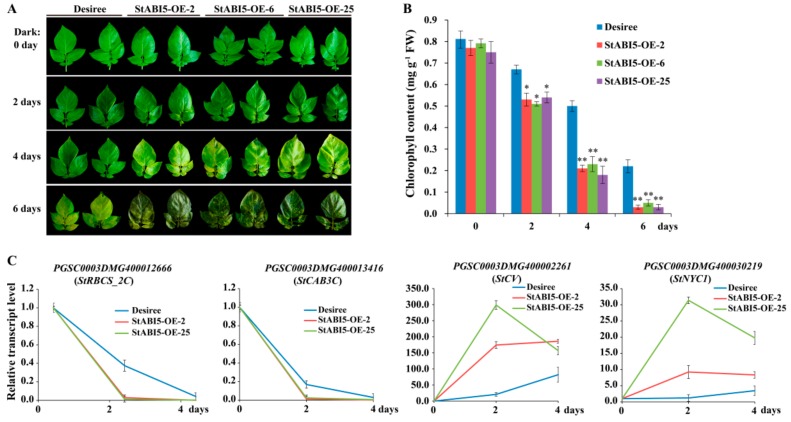
StABI5 regulates chlorophyll catabolism under dark conditions in potato. (**A**) Overexpressed *StABI5* (StABI5-OE) transgenic potato leaves accelerate yellowing and senescence under dark conditions. (**B**) Chlorophyll contents of StABI5-OE transgenic potato and Desiree leaves under dark conditions. The data represent an average of five leaves with three duplicates. Error bars indicate ±SD for triplicates. Asterisks denote Student’s *t*-test significant differences as compared with Desiree potatoes (* *P* <0.05; ** *P* < 0.01). (**C**) Transcription levels of chlorophyll metabolism-related genes *PGSC0003DMG400012666* (*StRBCS_2C*), *PGSC0003DMG400013416* (*StCAB3C*), *PGSC0003DMG400002261* (*StCV*) and *PGSC0003DMG400030219* (*StNYC1*) in StABI5-OE transgenic potato lines and Desiree leaves when treated with continuous darkness for 0, 2 and 4 days.

The overexpressed *P35S::StABI5-HA* (StABI5-OE) transgenic potato lines were planted in a field, and we found that StABI5-OE transgenic potato lines matured and aged significantly earlier than wild-type Desiree potatoes, and transgenic potato leaves showed phenotypes of yellowing and premature senescence (Figure S2), implying that StABI5 may participate in regulating chlorophyll metabolism. To verify whether StABI5 was involved in regulating chlorophyll metabolism, leaves of Desiree and StABI5-OE transgenic potato lines with similar phenotypes were treated with continuous darkness for 0, 2, 4 and 6 days, respectively. The results showed that StABI5-OE transgenic potato leaves accelerated yellowing and senescence under dark conditions (Figure 3A). Additionally, the chlorophyll contents of Desiree and StABI5-OE transgenic potato leaves were also measured. With an increase in darkness time, the chlorophyll contents of Desiree and StABI5-OE leaves were decreased, whereas StABI5-OE leaves showed a faster reduction in chlorophyll content (Figure 3B). These results indicated that StABI5 positively regulated chlorophyll catabolism under dark conditions in potato, which was consistent with the results in *Arabidopsis*.

To further test the effect of StABI5 on chlorophyll metabolism-related genes under dark conditions, we analyzed the transcription levels of chlorophyll metabolism-related genes by quantitative real-time PCR. *Chlorophyll a/b-binding protein 3* (*CAB3*) is a member of chlorophyll a/b-binding protein family, which encodes the most abundant CAB mRNA in developing embryos and young leaves [36]. Rubisco small subunit (RBCS) is a key rate-limiting enzyme in plant photosynthesis [37]. The transcription levels of *PGSC0003DMG400013416* (*StCAB3C*) and *PGSC0003DMG400012666* (*StRBCS_2C*) genes were significantly down-regulated by dark treatment. Moreover, the transcription levels of *StCAB3C* and *StRBCS_2C* displayed the highest down-regulation rate in StABI5-OE lines as compared to that observed in wild-type Desiree potatoes when treated with continuous darkness for 2 days (Figure 3C). The *AtCV* and *AtNYC1* genes positively regulate chloroplast degradation in *Arabidopsis* [15,38,39]. Consistent with the observations of *AtCV* and *AtNYC1* in *Arabidopsis*, the transcription levels of *PGSC0003DMG400002261* (*StCV*) and *PGSC0003DMG400030219* (*StNYC1*) genes were significantly up-regulated with dark treatment. However, the up-regulated rate of *StCV* and *StNYC1* genes in StABI5-OE lines was significantly higher than that of Desiree potatoes (Figure 3C). Interestingly, *StCV* and *StNYC1* genes contained conserved G-box element in their promoter regions, which implied that StABI5 binds to the promoters of the two genes to activate their expression. In addition, the expression levels of *StCV* and *StNYC1* genes reached the highest level in StABI5-OE lines with dark treatment for 2 days, while the expression levels of the two genes were slowly increased during dark treatment for 0-4 days in Desiree potatoes. These results indicated that darkness can activate the transcriptional activity of StABI5 and accelerate chlorophyll degradation by inducing the expression of chlorophyll catabolism-related genes, which in turn causes leaf yellowing and senescence.

### 2.3. Analysis of Differentially Expressed Genes (DEGs) Under Dark Conditions

**Figure 4:**
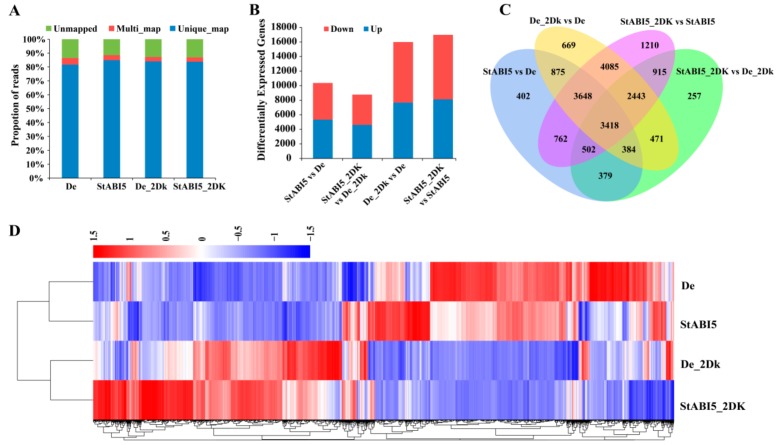
Analysis of differentially expressed genes. (**A**) Proportions of clean reads that were unmapped, mapped to multiple genes, and mapped to unique genes, which were plotted by three replicates of De, StABI5, De_2DK, and StABI5_2DK, respectively. (**B**) Statistical analysis of differentially expressed genes between different samples. (**C**) Analysis of the overlap of differentially expressed genes between different comparison combinations by Venn diagram. (**D**) Cluster analysis of differentially expressed genes by heat map. Red represents high gene abundance, blue represents low gene abundance.

To further verify the functions of StABI5, we performed transcriptome sequencing using overexpressed *StABI5* transgenic potato and wild-type Desiree leaves. According to previous experiments, we found that overexpressed *StABI5* transgenic potato line 25 (StABI5-OE-25) had the highest protein expression level. Furthermore, the transcription levels of chlorophyll catabolism-related genes reached the highest in StABI5-OE-25 line when treated with darkness for 2 days. Therefore, we chose the StABI5-OE-25 line as the best experimental material and 2 days of continuous darkness as the best time node for transcriptome sequencing. RNA-seq was conducted in StABI5-OE-25 and Desiree leaves treated with continuous darkness for 2 days, respectively. After filtering the raw data, checking the sequencing error rate, and calculating the GC content distribution, we finally got clean reads for subsequent analysis (Table 1). Approximately 87% of the reads can be mapped to the annotated *Solanum tuberosum* genome, and more than 81% of the reads were uniquely mapped to the genome in each sample (Figure 4A). Here, 10,370 differentially expressed genes (DEGs) were found in StABI5 vs. De group, of which 5324 DEGs were up-regulated and 5046 DEGs were down-regulated. An amount of 8769 DEGs were found in StABI5_2DK vs. De_2Dk group, of which 4616 DEGs were up-regulated and 4153 DEGs were down-regulated (Figure 4B). The Venn diagram displayed that 4683 DEGs overlapped between StABI5 vs. De and StABI5_2DK vs. De_2Dk. Here, 13,594 DEGs overlapped between De_2Dk vs. De and StABI5_2DK vs. StABI5, and 3418 DEGs overlapped among the four groups (Figure 4C). Furthermore, hierarchical cluster analysis of DEGs was performed using the Cluster software package. The results showed that the expression levels of many genes were changed in StABI5-OE-25 line as compared to Desiree potatoes (Figure 4D).

### 2.4. Gene ontology (GO) and KEGG Pathway Enrichment Analysis of DEGs

**Figure 5:**
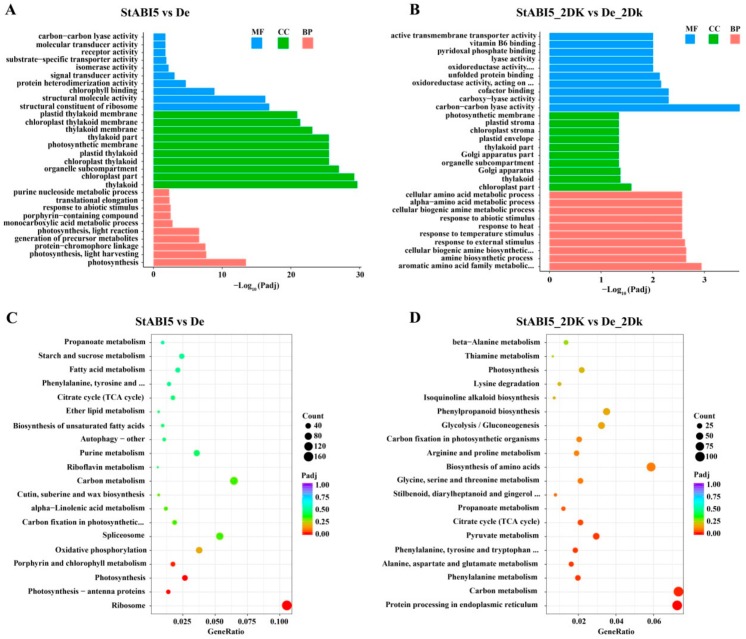
Gene ontology (GO) and KEGG pathway enrichment analysis of differentially expressed genes (DEGs). (**A**) The top 30 most enriched GO terms found in the analysis of DEGs in StABI5 vs. De group. Different colors represent molecular functions (MF), cellular components (CC), and biological processes (BP). Gene ontologies were ranked by their significance. (**B**) The top 30 most enriched GO terms found in the analysis of DEGs in StABI5_2DK vs. De_2Dk group. (**C**) The top 20 functionally enriched KEGG pathways found in the analysis of DEGs in StABI5 vs. De group. (**D**) The top 20 functionally enriched KEGG pathways found in the analysis of DEGs in StABI5_2DK vs. De_2Dk group.

To analyze the function of these DEGs and further understand the regulation of StABI5 in potato growth and development under dark conditions, we performed gene ontology (GO) assignments and enrichments. These DEGs were assigned to one or more of three categories: biological process, cellular component, and molecular function base on GO assignment, and they were significantly enriched in 153 and 113 terms of three GO categories in StABI5 vs. De and StABI5_2DK vs. De_2Dk groups, respectively (Supplementary Table S1). The most significant enrichment GO terms of the biological process were “photosynthesis” and “photosynthesis, light harvesting”, of the cellular component were “thylakoid” and “chloroplast part”, and those of the molecular function were “structural constituent of ribosome” and “chlorophyll binding” in StABI5 vs. De group (Figure 5A). Besides, the top three significantly enriched GO terms were “aromatic amino acid family metabolic process”, “chloroplast part” and “carbon-carbon lyase activity” in StABI5_2DK vs. De_2Dk group (Figure 5B), suggesting that StABI5 regulates multiple cellular processes in potato.

To provide further insight into metabolic pathways and signal transduction pathways, KEGG pathway analysis of DEGs was performed. The top three enriched KEGG pathways were “ribosome”, “photosynthesis-antenna proteins” and “photosynthesis” in StABI5 vs. De group (Figure 5C), of which “ribosome” and “photosynthesis” were the two most significantly down-regulated KEGG pathways, and “circadian rhythm-plant” was the most significantly up-regulated KEGG pathway. Additionally, the top three enriched KEGG pathways were “protein processing in endoplasmic reticulum”, “carbon metabolism” and “phenylalanine metabolism” in StABI5_2DK vs. De_2Dk group (Figure 5D). Collectively, these results and observations suggested that StABI5 was involved in regulating various metabolic pathways in potato, especially photosynthesis.

### 2.5. DEGs Involved in the Regulation of Chloroplast Development and Photosynthesis in Potato

Chloroplasts contain the green pigment chlorophyll and are responsible for the light- driven reactions of photosynthesis, upon which essentially all life depends [40]. Chloroplast biogenesis and development plays a key role in photosynthesis efficiency. Previous studies have shown that ABI5 is involved in regulating plant photosynthesis in *Arabidopsis*. AtABI5 directly inhibits *ABR* gene expression, thereby suppressing photosynthesis and accelerating leaf senescence [16]. The promoters of chlorophyll catabolic genes *SGR1* and *NYC1* contain ABRE elements, and ABI5 activates the expression of these genes by binding to the ABRE elements to promote chlorophyll degradation [15]. The RNA-seq analysis showed that chlorophyll catabolic genes *PGSC0003DMG400002261* (*StCV*) and *PGSC0003DMG400016833* (*StSGR1*) were up-regulated 1.75- and 2.57-fold in StABI5 vs. De group, respectively. Furthermore, most genes involved in “photosynthesis” and “carbon fixation”, such as chlorophyll a-b binding proteins, rubisco small subunits, and rubisco large subunits, were significantly down-regulated in StABI5 vs. De group (Table 2). Importantly, almost all genes of “chlorophyll a-b binding protein”, “photosystem I” and “photosystem II” were down-regulated, indicating that StABI5 negatively regulated the expression of photosynthesis-related genes. Taken together, the RNA-seq data explain the phenotype of leaf yellowing in StABI5-OE lines.

### 2.6. StABI5 Involved in the Regulation of Plant Hormone Signal Transduction in Potato

**Figure 6:**
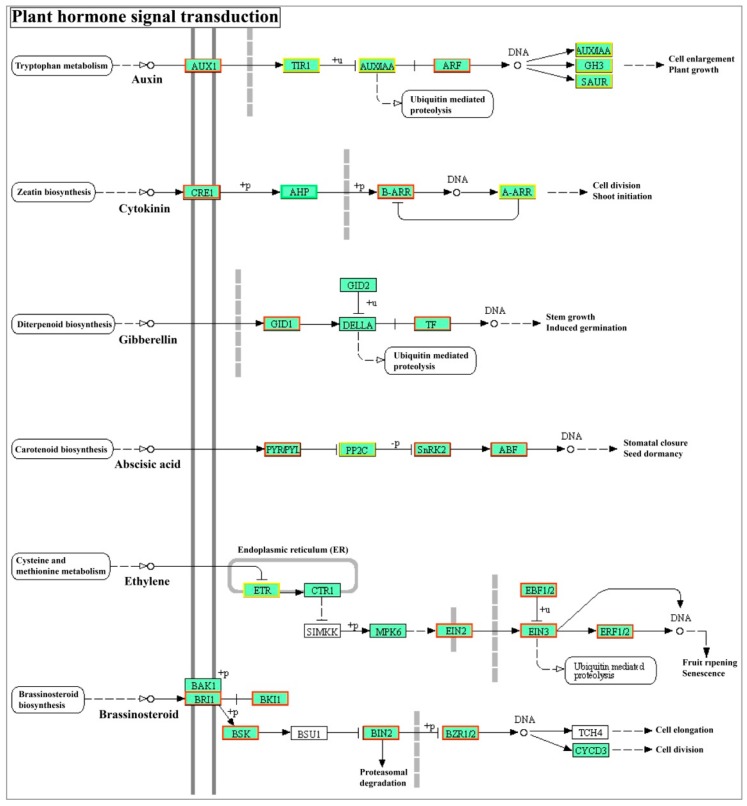
StABI5 involved in plant hormone signal transduction in potato. DEGs of plant hormone signals transduction were marked in StABI5 vs. De group. Green boxes represent down-regulated genes, red boxes indicate up-regulated genes, and yellow boxes represent both up- and down-regulated genes.

Plant hormones, including auxin, gibberellin (GA), cytokinin (CK), ABA, ethylene, and brassinosteroid, play important regulatory roles in plant growth and development, such as in seed germination, seedling growth, flowering, and fruit ripening. Recent studies indicated that ABI5 is involved in or regulated by these plant hormone signals [33,41,42]. Through the transcriptome data, we found that StABI5 was also involved in the regulation of plant hormone signaling in potato (Figure 6). Most genes related to the auxin pathway such as *SAUR family protein* and *AUX* were significantly changed, suggesting that StABI5 plays a role in the regulation of auxin signaling pathway (Table 3). In addition, the mRNA levels of genes associated with ABA, jasmonic acid, salicylic acid and brassinosteroid signals were up-regulated in StABI5 vs. De group. Importantly, all genes of *PYR*/*PYL*, *SnRK2*, and *ABF* were up-regulated, indicating that ABA pathway was activated in StABI5-OE-25 potato. Interestingly, we also found that most genes were up-regulated in the ethylene signaling pathway, especially *EIN2*/*3* and ethylene response factor *ERF1*/*2* genes, which were significantly up-regulated (Table 3), explaining the phenotype of leaves premature senescence in overexpressed *StABI5* transgenic potato lines. These data showed that StABI5 was involved in the regulation of plant hormone signals to balance potato growth and development.

### 2.7. Validation of Transcriptome Data by Quantitative Real-Time PCR

To confirm these transcriptome-based observations and the reliability of RNA-seq data, fifteen differentially expressed genes were randomly selected from the RNA-seq data in StABI5 vs. De group for quantitative real-time PCR. The changes in the transcription levels of 15 genes, including *PGSC0003DMG400002660* and *PGSC0003DMG400001390*, displayed the same trends as gained in RNA-seq data (Figure S3), indicating that the RNA-seq data were reliable and valid.

## 3. Discussion

Plants have evolved a set of precise molecular regulatory mechanisms in response to external biotic and abiotic stresses. ABA is one of the most important phytohormones to fight against environmental stresses in various plants [1,2,43]. It also has an essential role in multiple physiological processes of plants, such as seed germination, lateral root formation, leaf senescence, and stomatal closure [43,44]. In-depth dissection of the biological functions of essential regulatory proteins in the ABA signaling pathway is necessary for understanding physiological and metabolic processes involved in ABA signaling. The ABA signaling pathway is a complex signaling network that integrates with other signaling pathways including BR, CK, JA, GA and auxin signals. Many studies have shown that ABI5, as a key positive transcription factor in the ABA signaling pathway, not only responds to ABA to regulate the expression of ABA-responsive genes, but also participates in the regulation of other plant hormone signaling pathways [10,33,41,42]. Components of auxin, CK, GA, JA and BR signals were shown to take part in ABI5 regulation or to be regulated by ABI5 [10,33,41,42]. For example, ABI5 negatively regulates PIN1 accumulation and represses auxin activity in roots [45]. In this study, the transcriptome data results showed that mRNA levels of many genes involved in auxin, CK, GA, BR, ethylene and JA signaling pathways were changed in overexpressed *StABI5* transgenic potatoes, suggesting that StABI5 is also involved in regulating these plant hormone signaling pathways in potato.

Photosynthesis is a series of physiological and biochemical processes in chloroplasts. The ability of photosynthesis determines plant growth status, while chloroplast biogenesis and function affect the efficiency of photosynthesis. Recent studies have shown that photosynthesis is regulated by the target of rapamycin (TOR) and ABA signaling pathways [42,46,47]. TOR signaling acts as a positive regulatory factor to promote plant chloroplast biogenesis and photosynthesis, whereas ABA signaling acts as a negative regulatory signal to inhibit photosynthesis [42,47,48,49,50]. The transcription factor ABI4 of the ABA signaling pathway binds to the promoter regions of photosynthetic genes such as *LHCB* and *RBCS*, suppressing the expression of these genes and thus inhibiting photosynthesis [51,52]. Furthermore, ABI4 negatively regulates cotyledon greening and inhibits photosynthesis and seedling growth in *Arabidopsis* [50]. AtABI5 directly binds the promoter regions of chlorophyll catabolism genes *AtSGR1* and *AtNYC1* to activate the expression of the two genes in *Arabidopsis* [15,34,35], suggesting that AtABI5 negatively regulates photosynthesis by promoting chlorophyll degradation. Under dark conditions, AtABI5 inhibits the expression of *ABR* gene, accelerating leaf senescence [16]. Therefore, these studies show that ABI5 acts as a negative regulator of photosynthesis in *Arabidopsis*. In this study, we further verified that StABI5 was involved in regulating chloroplast development and photosynthesis in potato. In the dark, overexpressed *StABI5* transgenic potato leaves displayed accelerated yellowing and senescence phenotypes. The chlorophyll content between overexpressed *StABI5* transgenic potato lines and Desiree potatoes displayed a significant difference under dark conditions. Besides, we found that chlorophyll catabolic genes *StCV*, *StSGR1*, and *StNYC1* contained conserved G-box element in their promoter regions, and the mRNA levels of these genes were up-regulated in *StABI5* transgenic potato lines, showing that darkness activates the transcriptional activity of StABI5, which degrades chlorophyll by inducing the expression of chlorophyll catabolism-related genes. However, whether StABI5 directly binds to promoter regions of *StCV*, *StSGR1*, and *StNYC1* genes still requires further study.

Through analysis of the RNA-seq data, we found that many intracellular metabolic processes were changed, such as photosynthesis and carbon metabolism. Importantly, most of genes involved in “photosynthesis” and “carbon fixation” were significantly down-regulated in StABI5 vs. De group. Chlorophyll a-b binding proteins are components of photosynthetic antenna complexes and play vital roles in photosystems I and II [53]. The disruption of chlorophyll a-b binding proteins reduces photosynthesis efficiency [53,54]. The RNA-seq data showed that all genes of chlorophyll a-b binding proteins were down-regulated in overexpressed *StABI5* transgenic potatoes, indicating that StABI5 negatively regulates the expression of photosynthesis-related genes. Collectively, these observations, together with the findings in the RNA-seq data, suggested that StABI5 negatively regulates chloroplast development and photosynthesis in potato.

## 4. Materials and Methods

### 4.1. Plant Materials and Growth Conditions

Wild type *Arabidopsis* Columbia (Col) and Wassileskija (WS) seeds were used in this study. The *abi5-1* mutant seeds from the WS background were obtained from Dr. Zhizhong Gong. Wild-type Desiree potato seedlings were provided by Professor Jin Liping. The main overexpression vectors *P35S::AtABI5-HA*, *P35S::StABI5-HA*, and *PStABI5::StABI5-GFP* were used in this study. The *Arabidopsis* seeds were surface sterilized using liquid methods. The seeds were first treated with 70% ethanol for 2 min and then the supernatant was discarded. Then, the seeds were treated with 10% sodium hypochlorite, containing 0.3% Tween-20, for 5 min, and the supernatant was discarded. This was followed by rinsing four times with sterile water, centrifugation at 4000× *g* for 2 min each time, and the supernatant was discarded. Finally, the seeds were suspended in 0.15% sterile agarose and kept at 4 °C for 2 days. Sterilized seeds were plated on plates and grown in a growth chamber at 22 °C under 16 h of 60–80 µE· m^–2^ s^–1^ continuous light and 8 h of darkness.

### 4.2. Dark Treatment of Overexpression AtABI5 Transgenic Arabidopsis

The seeds of Col, WS, abi5-1 and overexpressed *AtABI5 Arabidopsis* seeds were sterilized and then placed on a 0.5 × MS solid medium for 10 days in a growth chamber at 22 °C under 16 h of continuous light and 8 h of darkness. Then the seedlings were transplanted to small flowerpots for 4 weeks under normal conditions. Seedlings of Col, WS, *abi5-1*, and overexpressed *AtABI5 Arabidopsis* were transferred to dark conditions for 8 days.

### 4.3. Dark Treatment of Arabidopsis and Potato Leaves

Seedlings of Col, WS, *abi5-1*, and overexpressed *AtABI5 Arabidopsis* were transplanted to small flowerpots for 4 weeks under normal conditions, and then the leaves of Col, WS, *abi5-1* and overexpressed *AtABI5 Arabidopsis* plants were placed in 6-well plates with sterile water for 8 days under dark conditions. Finally, the leaf phenotypes were observed.

Seedlings of Desiree and overexpressed *StABI5* (StABI5-OE) transgenic potato lines were grown in flowerpots for approximately 4 weeks at 22 °C under 16 h of continuous light and 8 h of darkness. Then, the leaves of the Desiree and StABI5-OE plants were cultured for 6 days in continuous darkness in a wet filter paper tray and the phenotypes of the leaves were observed.

### 4.4. Chlorophyll Content Measurement

The chlorophyll content of the *Arabidopsis* and potato leaves was measured for 0–8 days under dark conditions. Chlorophyll was extracted from the plant leaves and then quantified [55]. This was done by weighing 0.5 g samples in 15 mL centrifuge tubes, placing the tubes into a −20 °C refrigerator for 2 h. Then the tubes were taken out and 10 mL 80% acetone was added. The tubes were stored at 50 °C for 4 h in a dark cabinet. After mixing, chlorophyll content was determined photometrically by measuring absorption at 663 nm and 645 nm, and chlorophyll content was then calculated as described previously [16].

### 4.5. Total RNA Extraction and Detection for Transcriptome Sequencing

The leaves of the StABI5-OE-25 line and wild-type Desiree were treated with darkness for 0 and 2 days. Total RNA of the StABI5-OE-25 line and Desiree leaves, including those dark-treated 0 and 2 days, were isolated using the RNAprep Pure Plant Kit (TIANGEN, Beijing, China), respectively. For each treatment, three independent biological replicates were performed. RNA purity was checked using a NanoPhotometer^®^ spectrophotometer. RNA concentration was measured using Qubit^®^ RNA Assay Kit in Qubit^®^ 2.0 Flurometer. RNA integrity was assessed using the RNA Nano 6000 Assay Kit with the Bioanalyzer 2100 system.

### 4.6. RNA library Construction and Transcriptome Sequencing

A total amount of 3 µg RNA per sample was used as input material for the RNA sample preparations. Sequencing libraries were generated using NEBNext^®^ UltraTM RNA Library Prep Kit for Illumina^®^ (NEB, Ipswich, MA, USA) following the manufacturer’s recommendations and index codes were added to attribute sequences to each sample. After cluster generation, the library preparations were sequenced on an Illumina platform and 150 bp paired-end reads were generated.

### 4.7. Reads Mapping to the Reference Genome

Clean data (clean reads) were obtained by removing adapter, ploy-N, and low quality reads from the raw data. At the same time, the Q20, Q30 and GC content of the clean data were calculated. All the downstream analyses were based on the clean data with high quality. Reference genome and gene model annotation files were downloaded from the potato genome database website (http://plants.ensembl.org/Solanum_tuberosum/Info/Index). Index of the reference genome was built using Hisat2 (v2.0.5) and paired-end clean reads were aligned to the reference genome using Hisat2 (v2.0.5) [56].

### 4.8. Differential Expression Analysis

A differential expression analysis of two groups was performed using the DESeq2 R package (1.16.1) [57]. The resulting P-values were adjusted using the Benjamini and Hochberg’s approach for controlling the false discovery rate. Genes with an adjusted *P*-value (Padj) < 0.05 found by DESeq2 were assigned as differentially expressed genes (DEGs).

### 4.9. GO and KEGG Enrichment Analysis of Differentially Expressed Genes

A gene ontology (GO) enrichment analysis of DEGs was implemented by the clusterProfiler R package, where gene length bias was corrected. GO terms with corrected P-value < 0.05 were considered significantly enriched by differentially expressed genes. KEGG is a database resource for understanding high-level functions and utilities of biological systems, such as cells, organisms and ecosystems, from molecular level information, especially large-scale molecular datasets generated by genome sequencing and other high-through put experimental technologies. We used the clusterProfiler R package (with a corrected *P*-value < 0.05) to test the statistical enrichment of differentially expression genes in KEGG pathways.

### 4.10. Quantitative Real-Time PCR

Total RNA of both the StABI5-OE transgenic potato and Desiree leaves, including those treated with darkness for 0 and 2 days, was isolated using the RNAprep Pure Plant Kit (TIANGEN, Beijing, China). The PrimeScript^®^ RT reagent kit (Takara, Dalian, China) was used for reverse transcription following the manufacturer’s instructions. Relative transcription levels were assayed by two-step real-time PCR analysis using the CFX96 real-time PCR system (BIO-RAD, USA). Real-time primers were designed by Primer premier 5.0 and the details were presented in Supplementary Table S2. *StACTIN58* was used as an internal control. The data represent the mean ± SD of three independent experiments.

## Figures and Tables

**Table 1:** Summary of RNA-seq data.

Sample	Raw Reads	Clean Reads	Clean Bases	Error Rate (%)	Q20 (%)	Q30 (%)	GC Content (%)
De_1	51305060	49151244	7.37G	0.02	98.06	94.21	42.66
De_2	49674828	47359662	7.1G	0.02	98.21	94.57	42.83
De_3	58613332	56616148	8.49G	0.02	98.12	94.38	42.27
StABI5_1	58077052	56123334	8.42G	0.02	98.15	94.38	42.76
StABI5_2	68409014	66871468	10.03G	0.03	98.00	93.97	42.77
StABI5_3	67555414	65226846	9.78G	0.03	98.01	94.06	42.83
De_2Dk_1	54602684	52122618	7.82G	0.02	98.16	94.47	42.12
De_2Dk_2	60997616	56806266	8.52G	0.03	97.98	94.03	42.15
De_2Dk_3	51539718	49560170	7.43G	0.03	97.82	93.62	42.20
StABI5_2DK_1	56909932	54989466	8.25G	0.03	97.9	93.79	42.17
StABI5_2DK_2	66680642	64385132	9.66G	0.03	98.04	94.1	42.16
StABI5_2DK_3	76207430	74339060	11.15G	0.03	97.83	93.67	42.48

De: Desiree; StABI5: StABI5-OE-25; De_2Dk: Desiree treated with continuous darkness for 2 days; StABI5_2DK: StABI5-OE-25 treated with continuous darkness for 2 days.

**Table 2:** StABI5 involved in the regulation of photosynthesis in potato.

Gene_id	Log2FC	Padj	Gene_Description
**Photosynthesis—antenna proteins**			
PGSC0003DMG400007787	−1.91	5.51E-91	Chlorophyll a-b binding protein 8
PGSC0003DMG400006149	−1.60	7.63E-91	Chlorophyll a-b binding protein 4
PGSC0003DMG400016695	−1.83	4.07E-87	Chlorophyll a-b binding protein 50
PGSC0003DMG400004301	−1.84	3.29E-58	Chlorophyll a,b binding protein type I
PGSC0003DMG400042498	−1.64	7.88E-53	Chlorophyll a/b binding protein
PGSC0003DMG400013417	−1.57	2.23E-51	Chlorophyll a-b binding protein 3C
PGSC0003DMG400033084	−1.76	3.08E-48	Chlorophyll a/b-binding protein (cab-12)
PGSC0003DMG400008804	−1.47	2.13E-46	Chlorophyll a/b binding protein
PGSC0003DMG400026500	−1.18	1.97E-44	Type I (26 kD) CP29 polypeptide
PGSC0003DMG400023344	−1.16	9.28E-43	Chlorophyll a-b binding protein 6A
PGSC0003DMG400008298	−1.51	1.38E-41	Chlorophyll a/b binding protein
PGSC0003DMG400012590	−1.30	1.90E-40	Chlorophyll a-b binding protein CP24 10B
PGSC0003DMG400004458	−1.38	6.70E-38	Light-harvesting complex I protein Lhca5
PGSC0003DMG400019248	−1.12	2.95E-36	Chlorophyll a-b binding protein 13
PGSC0003DMG400008564	−1.09	3.60E-34	Chlorophyll a-b binding protein 13
PGSC0003DMG400012591	−1.08	6.93E-32	Chlorophyll a-b binding protein CP24 10A
PGSC0003DMG400008488	−0.90	4.29E-23	Chloroplast pigment-binding protein CP29
PGSC0003DMG400008309	−2.19	6.76E-21	Chlorophyll a/b binding protein
PGSC0003DMG400021287	−0.69	9.12E-17	Chlorophyll a-b binding protein 8
PGSC0003DMG400002901	−0.79	3.46E-16	Chlorophyll A/B binding protein
PGSC0003DMG401009929	0.72	3.57E-12	Light-harvesting complex II protein Lhcb7
PGSC0003DMG400023461	−0.48	5.67E-08	Chlorophyll a-b binding protein 6A
**Photosynthesis**			
PGSC0003DMG400004211	−1.84	4.83E-88	Photosystem Q(B) protein
PGSC0003DMG400005805	−1.53	3.51E-67	Photosystem I reaction center subunit
PGSC0003DMG400005890	−1.55	1.25E-60	16kDa membrane protein
PGSC0003DMG400007536	−1.37	8.99E-58	Photosystem II reaction center W protein
PGSC0003DMG400018434	−1.33	1.57E-56	Oxygen evolving enhancer protein 3
PGSC0003DMG400010035	−1.29	9.84E-56	Oxygen-evolving enhancer protein 1
PGSC0003DMG400020505	−1.53	1.33E-48	Photosystem I reaction center subunit X
PGSC0003DMG400000926	−1.15	1.40E-47	-
PGSC0003DMG400027672	−1.32	4.17E-46	Photosystem I subunit XI
PGSC0003DMG400021727	−1.15	7.78E-45	Photosystem II oxygen-evolving complex
PGSC0003DMG400008585	−1.24	9.79E-44	Photosystem II reaction center psb28 protein
PGSC0003DMG400026667	−1.23	7.16E-41	Isoform 2 of PsbP 2, chloroplastic
PGSC0003DMG400002626	−1.18	1.16E-40	Photosystem I psaH protein
PGSC0003DMG400022022	−1.09	6.81E-38	Photosystem I reaction center subunit IV B
PGSC0003DMG400027671	−1.11	2.60E-37	Photosystem I subunit XI
PGSC0003DMG400020141	−1.35	4.89E-35	Photosystem II reaction center W protein
PGSC0003DMG400007201	−1.04	3.06E-34	Photosystem II core complex proteins psbY
PGSC0003DMG400046303	−1.82	3.40E-34	Photosystem II CP47 chlorophyll apoprotein
PGSC0003DMG400002782	−0.90	2.78E-32	Oxygen-evolving enhancer protein 1
PGSC0003DMG400020466	−1.01	1.17E-31	ATP synthase subunit b’, chloroplastic
PGSC0003DMG400022249	−1.05	6.05E-31	Chloroplast photosystem I reaction center V
PGSC0003DMG400016504	−1.02	2.05E-29	PSI-H
PGSC0003DMG400016959	−0.99	2.44E-29	ATP synthase delta chain, chloroplastic
PGSC0003DMG400024531	−1.01	5.13E-27	Thylakoid lumenal 25.6 kDa protein
PGSC0003DMG400035711	−1.00	8.10E-27	Ferredoxin-1
PGSC0003DMG400007571	−0.94	1.14E-26	PSI-H
PGSC0003DMG400020484	−0.82	8.96E-25	ATP synthase subunit b’, chloroplastic
PGSC0003DMG400014402	−1.04	5.25E-22	Oxygen-evolving enhancer protein 3-1
PGSC0003DMG402003567	−0.69	6.32E-20	Ferredoxin--NADP reductase
PGSC0003DMG400021144	−0.90	8.05E-20	Photosystem I subunit III
PGSC0003DMG400017556	−0.76	2.01E-18	Photosystem II 22 kDa protein, chloroplastic
PGSC0003DMG400016482	−0.68	3.14E-18	ATP synthase gamma chain, chloroplastic
PGSC0003DMG400018360	−0.68	9.10E-14	Photosystem II 11 kDa protein
PGSC0003DMG400005372	−1.92	4.12E-13	Photosystem I P700 chlorophyll a apoprotein
PGSC0003DMG400011811	0.83	3.12E-10	Ferredoxin--NADP reductase
PGSC0003DMG400011816	−0.55	1.38E-09	Photosystem I reaction centre PSI-D subunit
PGSC0003DMG402005881	−0.95	5.17E-09	Ferredoxin-3, chloroplast
PGSC0003DMG400017532	−0.55	2.16E-08	Ferredoxin-2
PGSC0003DMG400026360	1.41	8.72E-05	Ferredoxin-3, chloroplast
PGSC0003DMG400010353	−0.66	0.000120	Cytochrome c6, chloroplastic
PGSC0003DMG400025106	−1.39	0.000357	ATP synthase epsilon chain, chloroplastic
PGSC0003DMG400023985	1.80	0.003149	Ferredoxin-3, chloroplast
PGSC0003DMG400002312	2.55	0.028061	Photosystem II 10 kDa polypeptide
**Carbon fixation in photosynthetic organisms**			
PGSC0003DMG400033037	−2.38	4.99E-82	Ribulose bisphosphate carboxylase large chain
PGSC0003DMG400012666	−1.66	1.70E-67	Ribulose bisphosphate carboxylase small chain 2C
PGSC0003DMG400019189	−1.56	5.21E-39	Fructose-1,6-bisphosphatase
PGSC0003DMG400019584	−1.00	2.18E-33	Ribulose bisphosphate carboxylase small chain 1
PGSC0003DMG400007466	1.41	2.03E-23	Phosphoenolpyruvate carboxylase
PGSC0003DMG400004436	−0.91	3.09E-21	Triosephosphate isomerase, chloroplastic
PGSC0003DMG400019188	−0.79	1.42E-19	Chloroplast fructose-1,6-bisphosphatase I
PGSC0003DMG400021264	0.87	7.73E-14	Phosphoenolpyruvate carboxylase
PGSC0003DMG400015385	0.75	2.84E-13	Phosphoenolpyruvate carboxylase
PGSC0003DMG400029406	−0.62	1.24E-12	Glyceraldehyde-3-phosphate dehydrogenase
PGSC0003DMG400033874	0.58	1.92E-11	Malic enzyme
PGSC0003DMG400003548	−0.46	2.2E-10	Fructose-bisphosphate aldolase
PGSC0003DMG400031063	0.47	1.38E-09	Malate dehydrogenase
PGSC0003DMG400011530	−0.50	9.18E-09	Glyceraldehyde-3-phosphate dehydrogenase
PGSC0003DMG400010788	−0.55	4.54E-08	Fructose-1,6-bisphosphatase, cytosolic
PGSC0003DMG400020416	0.71	9.46E-08	Aspartate aminotransferase
PGSC0003DMG400001595	−0.43	2.99E-07	Triosephosphate isomerase, chloroplastic
PGSC0003DMG400010840	0.76	3.88E-07	Aspartate aminotransferase
PGSC0003DMG400011246	1.10	4.86E-07	Glyceraldehyde-3-phosphate dehydrogenase
PGSC0003DMG400002675	0.58	9.16E-05	Fructose-bisphosphate aldolase
PGSC0003DMG400016094	1.91	3.39E-03	Phosphoenolpyruvate carboxykinase
PGSC0003DMG400015637	0.45	1.82E-02	Aspartate aminotransferase
PGSC0003DMG400011570	0.78	3.62E-02	NAD-malate dehydrogenase

**Table 3:** DEGs of plant hormone signals transduction in StABI5 vs. De group.

Gene_id	Log2FC	Padj	Gene_Description
**Auxin**			
PGSC0003DMG400006550	0.39	4.77E-02	Auxin influx transport protein
PGSC0003DMG400024033	0.45	6.28E-04	Transport inhibitor response 1
PGSC0003DMG400022404	−0.38	3.84E-05	F-box family protein
PGSC0003DMG400019302	−1.21	3.99E-04	Nt-iaa4.5 deduced protein
PGSC0003DMG400016317	−0.39	7.77E-03	LEAUX= auxin-regulated protein homolog
PGSC0003DMG400002608	1.57	3.16E-10	ARF domain class transcription factor
PGSC0003DMG400002392	0.94	1.8E-20	Auxin response factor 7
PGSC0003DMG400020711	0.84	1.5E-21	Auxin response factor 1
PGSC0003DMG400031769	0.56	3.95E-08	Auxin response factor 3
PGSC0003DMG400025856	1.64	7.91E-04	Auxin and ethylene responsive GH3
PGSC0003DMG400024995	−2.16	1.88E-04	Indole-3-acetic acid-amido synthetase GH3.6
PGSC0003DMG400019274	3.10	1.32E-44	Indole-3-acetic acid-amido synthetase GH3.6
PGSC0003DMG400024997	0.84	3.44E-14	Indole-3-acetic acid-amido synthetase GH3.6
PGSC0003DMG400001605	−2.07	3.18E-02	Conserved gene of unknown function
PGSC0003DMG400001614	−1.26	1.81E-12	SAUR family protein
PGSC0003DMG400001668	−1.26	9.96E-05	SAUR family protein
PGSC0003DMG400001667	−0.68	5.17E-09	SAUR family protein
PGSC0003DMG400001601	2.54	2.23E-02	Auxin-responsive family protein
PGSC0003DMG400026709	1.15	1.10E-02	SAUR family protein
**Cytokinin**			
PGSC0003DMG400029463	0.31	2.90E-02	Cytokinin receptor 1
PGSC0003DMG400018579	−1.44	7.55E-03	Histidine phosphotransfer protein
PGSC0003DMG400003196	1.12	5.04E-12	Two-component system sensor histidine kinase/response regulator
PGSC0003DMG400016643	2.32	1.33E-08	Two-component system sensor histidine kinase/response regulator
PGSC0003DMG400007823	0.42	3.71E-03	Two-component system sensor histidine kinase/response regulator
PGSC0003DMG400023534	0.60	3.10E-07	Type-B response regulator
PGSC0003DMG401025624	2.00	6.59E-20	Type-B response regulator
PGSC0003DMG400027597	−2.45	8.96E-03	Type-a response regulator
PGSC0003DMG400029852	−1.25	9.99E-08	Type-a response regulator
**Gibberellin**			
PGSC0003DMG400021991	1.49	3.54E-18	GID1-like gibberellin receptor
PGSC0003DMG400003849	1.33	5.66E-09	GID1-like gibberellin receptor
PGSC0003DMG401015926	0.93	3.25E-21	Isoform 2 of Transcription factor PIF5
**Abscisic acid**			
PGSC0003DMG400011033	0.71	4.54E-08	Abscisic acid receptor PYL4
PGSC0003DMG400029952	1.01	1.90E-02	Bet v I allergen family protein
PGSC0003DMG400015897	0.89	2.79E-05	Abscisic acid receptor PYL4
PGSC0003DMG400012155	0.40	1.45E-03	Pathogenesis-induced protein
PGSC0003DMG400029194	1.94	1.21E-02	Abscisic acid receptor PYL4
PGSC0003DMG400009112	-0.96	3.52E-02	Protein phosphatase 2C
PGSC0003DMG400002573	0.41	4.73E-05	Protein phosphatase 2C ABI2 homolog
PGSC0003DMG400016742	1.17	2.03E-03	Protein phosphatase 2C AHG3 homolog
PGSC0003DMG400029297	0.64	1.16E-08	Abscisic insensitive 1B
PGSC0003DMG400025895	1.07	2.62E-15	Serine/threonine-protein kinase SAPK10
PGSC0003DMG400029774	0.63	1.31E-03	Basic-leucine zipper
PGSC0003DMG400008011	0.38	2.27E-02	ABRE binding factor
PGSC0003DMG400002660	11.15	3.63E-63	DNA binding protein, ABI5
**Ethylene**			
PGSC0003DMG400031819	−0.41	2.39E-06	Ethylene receptor homolog
PGSC0003DMG400007843	0.54	3.61E-04	Ethylene receptor 1
PGSC0003DMG400028694	0.74	2.42E-09	Ethylene receptor homolog
PGSC0003DMG400016284	1.05	3.13E-11	Ethylene receptor
PGSC0003DMG400023402	0.89	2.04E-09	Ethylene receptor
PGSC0003DMG400021547	0.55	1.99E-09	Ethylene signaling protein
PGSC0003DMG400015853	0.61	1.7E-12	EIN3-binding F-box protein 1
PGSC0003DMG400030928	0.55	5.97E-06	EIN3-binding F-box protein 1
PGSC0003DMG400002914	1.05	1.41E-27	EIN3-binding F-box protein 1
PGSC0003DMG400008712	0.37	5.48E-03	EIL2
PGSC0003DMG400017231	1.49	1.09E-04	Transcription factor TSRF1
PGSC0003DMG400014204	1.85	2.26E-02	Transcription factor TSRF1
PGSC0003DMG400014594	2.01	9.78E-13	Ethylene response factor
**Brassinosteroid**			
PGSC0003DMG400019698	0.38	2.41E-04	BRI1 protein
PGSC0003DMG400015442	0.52	6.35E-05	Conserved gene of unknown function
PGSC0003DMG400009972	1.08	1.74E-23	Receptor protein kinase
PGSC0003DMG400001670	0.29	2.38E-03	Receptor protein kinase
PGSC0003DMG400026744	0.80	7.13E-11	Glycogen synthase kinase-3 beta
PGSC0003DMG400028428	0.55	8.79E-12	Glycogen synthase kinase-3 beta
PGSC0003DMG400004501	0.98	5.83E-16	BRASSINAZOLE-RESISTANT 1 protein
**Jasmonic acid**			
PGSC0003DMG400032119	0.48	1.00E-05	Jasmonate ZIM-domain protein 3
PGSC0003DMG400002930	1.96	2.56E-21	Jasmonate ZIM-domain protein 1
PGSC0003DMG400022888	0.76	1.17E-09	Salt responsive protein 1
PGSC0003DMG400029237	0.59	1.34E-05	Gene of unknown function
PGSC0003DMG400015667	0.36	2.63E-02	Pto-responsive gene 1 protein
**Salicylic acid**			
PGSC0003DMG401000923	1.79	1.02E-47	NIM1 2
PGSC0003DMG400021210	1.09	1.05E-26	NIM1 1
PGSC0003DMG400008160	1.26	3.48E-03	BOP/NPR1/NIM1-like regulatory protein

## Supplementary Materials

Supplementary materials can be found at https://www.mdpi.com/1422-0067/21/3/1068/s1. **Figure S1.** Bioinformatics analysis of StABI5. **(A)** Basic information about StABI5 on the *Solanum tuberosum* genome database website. **(B)** Phylogenetic tree analysis of ABI5 protein in *Solanum tuberosum* and other species. St: *Solanum tuberosum*, Sl: *Solanum lycopersicum*, Nt: *Nicotiana tabacum*, At: *Arabidopsis thaliana*, and Os: *Oryza sativa*. **(C)** Conserved domains analysis of StABI5. **Figure S2.** The phenotype of StABI5-OE transgenic potato lines planted in the field for one month. **Figure S3.** Validation of transcriptome data by qRT-PCR. **Supplementary Table S1.** Significantly enriched Go terms. **Supplementary Table S2.** Primers for quantitative real-time PCR in this study.
